# Clinical implications of using both fluoropyrimidine and paclitaxel in patients with severe peritoneal metastasis of gastric cancer: A post hoc study of JCOG1108/WJOG7312G

**DOI:** 10.1002/cam4.4303

**Published:** 2021-10-16

**Authors:** Hiroyuki Arai, Eisuke Inoue, Kensei Yamaguchi, Narikazu Boku, Hiroki Hara, Tomohiro Nishina, Masahiro Tsuda, Kohei Shitara, Katsunori Shinozaki, Shinichiro Nakamura, Ichinosuke Hyodo, Kei Muro, Mitsuru Sasako, Masanori Terashima, Takako E. Nakajima

**Affiliations:** ^1^ Department of Clinical Oncology St. Marianna University School of Medicine Kawasaki Japan; ^2^ Norris Comprehensive Cancer Center University of Southern California Los Angeles USA; ^3^ Showa University Research Administration Center Showa University Tokyo Japan; ^4^ Department of Gastroenterology The Cancer Institute Hospital of Japanese Foundation for Cancer Research Tokyo Japan; ^5^ Department of Gastrointestinal Medical Oncology National Cancer Center Hospital Tokyo Japan; ^6^ Department of Gastroenterology Saitama Cancer Center Saitama Japan; ^7^ Department of Gastrointestinal Medical Oncology National Hospital Organization Shikoku Cancer Center Matsuyama Japan; ^8^ Department of Gastroenterological Oncology Hyogo Cancer Center Akashi Japan; ^9^ Gastroenterology and Gastrointestinal Oncology National Cancer Center Hospital East Kashiwa Japan; ^10^ Division of Clinical Oncology Hiroshima Prefectural Hospital Hiroshima Japan; ^11^ West Japan Oncology Group (WJOG) Data Center Division Yokohama Japan; ^12^ Department of Gastroenterology University of Tsukuba Tsukuba Japan; ^13^ Department of Clinical Oncology Aichi Cancer Center Nagoya Japan; ^14^ Department of Surgery Yodogawa Christian Hospital Osaka Japan; ^15^ Division of Gastric Surgery Shizuoka Cancer Center Nagaizumi Japan; ^16^ Kyoto Innovation Center for Next Generation Clinical Trials and iPS Cell Therapy (Ki‐CONNECT) Kyoto University Hospital Kyoto Japan

**Keywords:** FLTAX, gastric cancer, inadequate oral intake, massive ascites, severe peritoneal metastasis

## Abstract

**Background:**

In the JCOG1108/WJOG7312G trial, a combination (FLTAX) of 5‐fluorouracil (FU) /leucovorin (FL) and paclitaxel (PTX) did not show superiority in overall survival (OS) to FL in untreated patients with severe peritoneal metastasis of gastric cancer (GC‐SPM), some of whom received second‐line chemotherapy with PTX after FL. This post hoc study aimed to investigate the clinical implications of using both FU and PTX either sequentially or in combination for patients with GC‐SPM.

**Methods:**

A total of 94 patients were enrolled and categorized into the following three subgroups: patients treated with (1) FL followed by PTX (FL/PTX, *N *= 25), (2) FL followed by best supportive care (BSC) (FL/BSC, *N* = 21), and (3) FLTAX (*N* = 48). OS was compared between the subgroups. By comparing baseline factors between the FL/PTX and FL/BSC subgroups, factors preventing the sequential use of PTX (SUP) were explored using logistic regression model. The efficacy of FL and FLTAX was compared according to the presence of risk factors preventing SUP.

**Results:**

The FL/PTX subgroup showed better and equivalent OS compared to the FL/BSC (median 7.8 vs. 2.0 months, *p* < 0.01) and FLTAX (median 7.8 vs. 8.0, *p* = 0.49) subgroups, respectively. Glasgow Prognostic Score 2 and initially unresectable disease were identified as risk factors preventing SUP. Absence of both risks predicted SUP with a sensitivity of 13% and a specificity of 100%, whereas absence of any risks predicted SUP with a sensitivity of 67% and a specificity of 62%. FLTAX showed better OS than FL in patients with one or two of these risks but worse OS in those with none.

**Conclusions:**

Although sequential use of FU and PTX showed equivalent survival to FLTAX in patients with GC‐SPM, FLTAX might be preferable given the difficulty in selecting patients likely to receive sequential use at the initiation of first‐line chemotherapy.

## Graphical abstract

Using both fluoropyrimidine and paclitaxel, either sequentially or in combination, might contribute to a better prognosis in patients with severe peritoneal metastasis of gastric cancer. The preference of a combination regimen FLTAX was highlighted because patient selection for sequential use of both drugs was difficult.

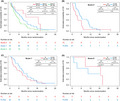

## 1 INTRODUCTION

Approximately 30%–50% of patients with recurrent and metastatic gastric cancer (GC) present with peritoneal metastasis (PM).^1^, ^2^, ^3^ Accordingly, studies have showed that PM is a poor prognostic factor that can promote rapid deterioration of the patient's condition.^4^, ^5^ Unfortunately, patients with severe PM (SPM), who present with massive ascites and/or bowel obstruction, find it hard to receive standard chemotherapies for advanced GC, such as a combination of S‐1 and cisplatin, given the need for stable oral intake and adequate intravenous hydration. Considering that the patients with SPM have been completely excluded from previous clinical trials on GC, they remain a niche subgroup of patients with unmet needs among whom effective and safe treatment strategies have yet to be developed.^6^, ^7^, ^8^, ^9^ Among the considerably few available therapeutic options, a feasible monotherapy with fluoropyrimidine (FU), such as the 5‐FU/*l*‐leucovorin (FL) regimen, has been used as the standard first‐line chemotherapy for patients with SPM of GC (GC‐SPM) in Japan. However, some retrospective studies have shown very modest efficacy, reporting a median overall survival (OS) of only 4.6–6.0 months.^10^, ^11^ Moreover, patients with GC‐SPM are less likely to receive second‐line treatment owing to rapid deterioration in their condition after FL failure, resulting in a missed chance to use paclitaxel (PTX)—another active medication for PM.^10^, ^11^, ^12^

JCOG1108/WJOG7312G, the first phase II/III trial in untreated patients with GC‐SPM, investigated the efficacy and safety of PTX plus FL (FLTAX) compared to FL.^13^ The aforementioned trial had been conducted based on the assumption that FLTAX was superior to FL given that the combination regimen would allow all patients to receive both FU and PTX as the first‐line treatment. Interestingly, their results revealed that FLTAX‐treated patients had a favorable but not significantly better OS compared to FL‐treated patients [median OS, 7.3 vs. 6.1 months; hazard ratio (HR) 0.79, 80% confidence interval (CI) 0.60–1.05; *p* = 0.14]. As expected, however, only a half of the FL‐treated patients could sequentially receive paclitaxel as their second‐line treatment.^13^ Given the aforementioned results, it still remains unclear^1^ whether using both FU and PTX, either sequentially or in combination, contributes to a better prognosis in patients with GC‐SPM^2^; what risk factors prevent sequential use of PTX (SUP) after FL failure; and^3^ which treatment strategy is preferable for using both drugs, in combination (FLTAX) or sequentially (FL followed by PTX). To address these clinical concerns, this post hoc study of the JCOG1108/WJOG7312G trial was conducted based on information regarding the participants’ first‐ and second‐line treatments.

## 2 METHODS

### 2.1 Summary of the JCOG1108/WJOG7312G trial

JCOG1108/WJOG7312G is a randomized phase II/III trial conducted across 43 institutions in Japan. The main inclusion criteria were histologically confirmed gastric adenocarcinoma; initially unresectable or recurrent disease; age 20–75 years; Eastern Cooperative Oncology Group performance status 0–2; having SPM defined as massive ascites throughout the abdominal cavity and/or inadequate oral intake requiring an intravenous drip infusion; and previously untreated disease. Eligible patients were randomly assigned at a 1:1 ratio to receive FL or FLTAX. The primary endpoint of the phase III portion was OS, whereas the main secondary endpoints included progression‐free survival (PFS), incidences of adverse events, improvement rate of oral intake, and ascites response rate. Although an initial sample size of 330 had been planned, the protocol was amended after 102 patients had been enrolled in the phase II portion due to poor accrual, the data of whom would be used for the final analysis in the phase III portion based on the amended statistical hypothesis. Full details regarding the study, such as treatment schedules, ethics committee approval, Declaration of Helsinki accordance, and main results, are reported elsewhere.^13^

### 2.2 Subject of this study

**FIGURE 1:**
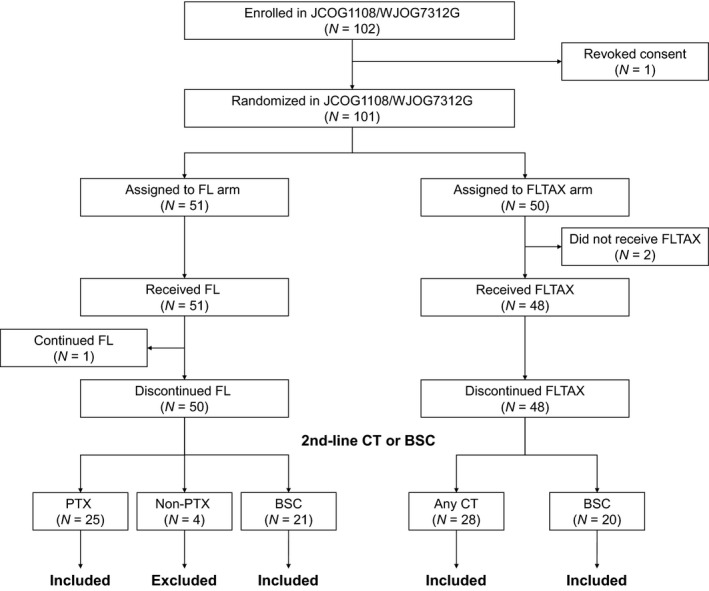
CONSORT diagram. Abbreviations: BSC, best supportive care; CT, chemotherapy; PTX, paclitaxel.

After excluding four patients (one withdrew consent before randomization; two did not receive FLTAX; and one was still receiving FL treatment at the last follow‐up date) from the 102 patients enrolled in the JCOG1108/WJOG7312G, 98 patients were categorized into the following four subgroups according to the use of FU and PTX during first‐ and second‐line treatments: (1) patients treated with FL followed by PTX (FL/PTX, *N* = 25); (2) those treated with FL followed by chemotherapy other than PTX (FL/non‐PTX, *N* = 4); (3) those treated with FL followed by best supportive care (BSC) (FL/BSC, *N* = 21); and (4) those treated with FLTAX followed by any chemotherapy (*N* = 28) or BSC (*N* = 20) (FLTAX, *N* = 48). Given that the JCOG1108/WJOG7312G trail did not plan to collect data beyond second‐line treatment, the four patients initially categorized into the FL/non‐PTX subgroup were excluded considering that it was impossible to evaluate the clinical significance of using both FL and PTX. Ultimately, 94 patients belonging to either FL/PTX, FL/BSC, or FLTAX subgroups were included herein (Figure 1).

### 2.3 Statistical methods

The FL/PTX and FL/BSC subgroups and FL/PTX and FLTAX subgroups were compared according to patient characteristics at baseline, OS, and PFS. OS was defined as the duration from the date of randomization to the date of death from any cause. PFS was defined as the duration from the date of randomization to the date of disease progression after first‐line treatment or death from any cause. Patients who had no events were censored at the last follow‐up date. Both OS and PFS were estimated using the Kaplan–Meier method, whereas differences therein were determined using the log‐rank test. HR and 95% CI were estimated using unadjusted Cox proportional hazards models. Categorical variables between the groups were compared using the chi‐squared test.

To identify the risk factors preventing SUP after FL failure, the FL/PTX and FL/BSC subgroups were compared according to each baseline factor using unadjusted logistic regression models, after which factors with a *p* value <0.20 were identified as risk factors preventing SUP. The number of the risk factors preventing SUP was used to indicate the SUP risk score. The predictive performance of the SUP risk score was assessed using sensitivity, specificity, positive predictive value (PPV), and negative predictive value (NPV) for SUP after FL. Finally, differences in treatment efficacy in terms of OS between the FL and FLTAX subgroups stratified according to the SUP risk score were determined. The log‐rank test and unadjusted Cox proportional hazards models were utilized to compare OS between the groups. All tests were two‐sided, with a *p* value <0.05 indicating statistical significance. All statistical analyses were conducted using the R version 4.0 statistical software (https://www.R‐project.org/).

## 3 RESULTS

### 3.1 Patient characteristics at baseline

Table 1 summarizes the baseline characteristics of each patient subgroup. The FL/BSC subgroup had a greater frequency of initially unresectable diseases compared to the FL/PTX subgroup (95% vs. 76%, *p* = 0.07).

**TABLE 1:** Patient characteristics at baseline.

		FL/PTX (*N* = 25)		FL/BSC (*N* = 21)		FLTAX (*N* = 48)		*p* value FL/PTX vs. FL/BSC	*p* value FL/PTX vs. FLTAX
		*N*	(%)	*N*	(%)	*N*	(%)		
Age	>65	10	(40)	11	(52)	26	(54)	0.40	0.25
	<65	15	(60)	10	(48)	22	(46)		
Sex	Male	16	(64)	12	(57)	30	(63)	0.64	0.90
	Female	9	(36)	9	(43)	18	(38)		
ECOG‐PS	0	4	(16)	2	(10)	7	(15)	0.69	0.96
	1	15	(60)	12	(57)	28	(58)		
	2	6	(24)	7	(33)	13	(27)		
GPS	0	5	(20)	2	(10)	11	(23)	0.29	0.48
	1	9	(36)	5	(24)	14	(29)		
	2	10	(40)	14	(67)	23	(48)		
	Unknown	1	(4)	0	(0)	0	(0)		
Disease status	Recurrent	6	(24)	1	(5)	7	(15)	0.07	0.32
	Initially unresectable	19	(76)	20	(95)	41	(85)		
No. of metastatic sites	1–2	21	(84)	17	(81)	38	(79)	0.79	0.62
	>3	4	(16)	4	(19)	10	(21)		
Histological type	Differentiated	5	(20)	6	(29)	5	(10)	0.40	0.33
	Undifferentiated	20	(80)	14	(67)	41	(85)		
	Others	0	(0)	1	(5)	2	(4)		
Massive ascites	Present	17	(68)	16	(76)	30	(63)	0.54	0.64
	Absent	8	(32)	5	(24)	18	(38)		
Oral intake	Adequate	14	(44)	11	(52)	22	(46)	0.81	0.41
	Inadequate	11	(56)	10	(48)	26	(54)		
Subtype of SPM	Massive ascites	14	(56)	11	(52)	22	(46)	0.55	0.70
	Inadequate oral intake	8	(32)	5	(24)	18	(38)		
	Both	3	(12)	5	(24)	8	(17)		

Abbreviations: BSC, best supportive care; ECOG‐PS, Eastern Cooperative Oncology Group performance status; GPS, Glasgow Prognostic Score; SPM, severe peritoneal metastasis.

### 3.2 Comparison of survival time between the FL/PTX and other subgroups

Patients in the FL/PTX subgroup showed better OS (median OS, 7.8 vs. 2.0 months; HR 0.24, 95% CI 0.12–0.48; log‐rank *p *< 0.01) and PFS (median PFS, 3.3 vs. 1.5 months; HR 0.54, 95% CI 0.30–0.99; log‐rank *p* = 0.04) compared to those in the FL/BSC subgroup (Figure S1A, B). Patients in the FL/PTX subgroup showed similar OS (median OS, 7.8 vs. 8.0 months; HR 0.83, 95% CI 0.50–1.39; log‐rank *p* = 0.49) but worse PFS (median PFS, 3.3 vs. 5.7 months; HR 1.74, 95%CI 1.05–2.87; log‐rank *p* = 0.03) compared to those in the FLTAX subgroup (Figure S1C, D).

### 3.3 Risk factors preventing SUP and predictive performance of the SUP risk score

Logistic regression analysis of the FL/PTX and FL/BSC subgroups identified two risk factors preventing SUP: GPS2 with an odds ratio (OR) of 0.29 (95% CI 0.05–1.78, *p* = 0.18) and 0.40 (95% CI 0.10–1.55, *p* = 0.18) compared to GPS0 and 1, respectively, and initially unresectable disease with an OR of 0.16 (95% CI 0.02–1.44, *p* = 0.10) compared to recurrent disease (Table 2). Thereafter, the SUP risk score was determined as follows: score of 2, presence of both GPS2 and initially unresectable disease; score of 1, presence of either; and score of 0, presence of neither. Accordingly, the sensitivity, specificity, PPV, and NPV of the SUP risk score were 13%, 100%, 100%, and 50%, respectively, using a score cutoff of 0 and 67%, 62%, 67%, and 62%, respectively, using a score cutoff of 1 (Table 3).

**TABLE 2:** Logistic regression analysis for likelihood of SUP after failure of FL.

Variable		Odds ratio	(95% CI)	*p* value
Age	<65 vs. >65	1.65	(0.51–5.33)	0.40
Sex	Male vs. Female	1.33	(0.41–4.38)	0.64
ECOG‐PS	1 vs. 0	0.63	(0.10–4.01)	0.62
	2 vs. 0	0.43	(0.06–3.22)	0.41
GPS	1 vs. 0	0.72	(0.10–5.17)	0.74
	2 vs. 0	0.29	(0.05–1.78)	**0.18**
	2 vs. 1			**0.18**
Disease status	Initially unresectable vs. Recurrent	0.16	(0.02–1.44)	**0.10**
No. of metastatic sites	>3 vs. 1–2	0.81	(0.18–3.72)	0.79
Histological type	Undifferentiated vs. Differentiated	1.71	(0.44–6.74)	0.44
Massive ascites	Absent vs. Present	1.51	(0.41–5.58)	0.54
Oral intake	Inadequate vs. Adequate	0.86	(0.27–2.77)	0.81
Subtype of SPM	Both vs. Massive ascites	0.47	(0.09–2.42)	0.37
	Inadequate oral intake vs. Massive ascites	1.26	(0.32–4.94)	0.74

Each variable was compared between the FL/PTX (*N* = 25) and FL/BSC (*N* = 21) subgroups using the logistic regression model. The odds ratio indicates each factor's impact on the likelihood of using second‐line paclitaxel after failure of first‐line FL. *P* values less than 0.20 are indicated in bold characters.

Abbreviations: CI, confidence interval; ECOG‐PS, Eastern Cooperative Oncology Group performance status; GPS, Glasgow Prognostic Score; SPM, severe peritoneal metastasis; SUP, sequential use of paclitaxel.

**TABLE 3:** Predictive performance of the SUP risk score in assessing SUP after failure of FL.

	SUP risk score 0	SUP risk score 0–1
True positive (*N*)	3	16
False positive (*N*)	0	8
True negative (*N*)	21	13
False negative (*N*)	21	8
Sensitivity	13%	67%
Specificity	100%	62%
PPV	100%	67%
NPV	50%	62%

Abbreviations: NPV, negative predictive value; PPV, positive predictive value; SUP, sequential use of paclitaxel.

### 3.4 Comparison of OS between FL and FLTAX stratified according to the SUP risk score

**FIGURE 2:**
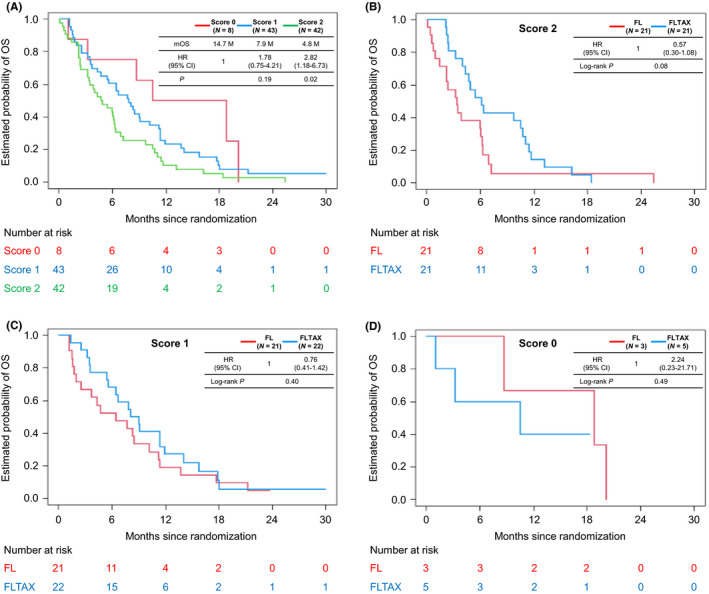
Kaplan–Meier curves according to the SUP risk score. Scores of 2, 1, and 0 indicate the presence of both GPS2 and initially unresectable disease, either, and neither, respectively. (A) OS comparing scores of 2 versus 0 and 1 versus 0 in both treatments; (B) OS comparing FL versus FLTAX in patients with a score of 2; (C) OS comparing FL versus FLTAX in those with a score of 1; and (D) OS comparing FL versus FLTAX in those with a score of 0. Abbreviations: CI, confidence interval; GPS, Glasgow Prognostic Score; HR, hazard ratio; mOS, median overall survival; OS, overall survival; SUP, sequential use of paclitaxel.

Among the 94 patients included herein, 42 (21 received FL and 21 received FLTAX) had a SUP risk score of 2, 43 (21 received FL and 22 received FLTAX) had a SUP risk score of 1, 8 (3 received FL and 5 received FLTAX) had a SUP risk score of 0, and one lacked data on GPS. Patients who received FL and FLTAX had similar patient characteristics at baseline stratified according to the SUP risk score. However, among those with a SUP risk score of 2, those receiving FLTAX had a higher proportion of males compared to those receiving FL (Table S1). Median OS in the patients with a SUP risk score of 2, 1, and 0 was 4.8, 7.9, and 14.7 months, respectively (SUP risk score of 2 vs. 0, HR 2.82, 95% CI 1.18–6.73, *p* = 0.02; SUP risk score of 1 vs. 0, HR 1.78, 95% CI 0.75–4.21, *p* = 0.19) (Figure 2A). Compared to FL, FLTAX showed a better OS in patients with a SUP risk score of 2 (HR 0.57, 95% CI 0.30–1.08, log‐rank *p* = 0.08) and 1 (HR 0.76, 95% CI 0.41–1.42, log‐rank *p* = 0.40) but worse OS in those with a SUP risk score of 0 (HR 2.24, 95% CI 0.23–21.71, log‐rank *p* = 0.49) (Figure 2B, C, D).

## 4 DISCUSSION

The current post hoc study of the JCOG1108/WJOG7312G trial explored the clinical implications of using both FU and PTX during first‐ and second‐line treatments in patients with GC‐SPM. Accordingly, our results showed that the FL/PTX and FLTAX subgroups exhibited better survival observed compared to the FL/BSC subgroup, indicated that using both drugs, either sequentially or in combination, may contribute to a better prognosis. However, this study did identify some baseline factors, such as GPS2 and initially unresectable disease that may decrease the chances of receiving PTX after FL failure, leading to a poor prognosis. Given the difficulty of selecting patients suitable for the sequential strategy at the initiation of the first‐line treatment, FLTAX has been considered the preferable combination regimen for the first‐line treatment of GC‐SPM.

Paclitaxel has been utilized as an active medication for the treatment of PM given that its high molecular weight, bulky structure, and protein‐binding affinity can promote extraordinarily low clearance from the intraperitoneal cavity, especially in the presence of malignant ascites wherein proteins exist at high concentrations.^14^, ^15^ The JCOG0407 trial showed that PTX had better efficacy compared to the best available 5‐FU regimen as a second‐line treatment for patients with PM of GC.^16^ Moreover, a retrospective study reported that taxane demonstrated promising efficacy and tolerable toxicities as a second‐line treatment for patients with GC‐SPM.^17^ The aforementioned evidences support the results presented herein showing better survival outcomes in patients who received sequential PTX compared to those who underwent BSC after the failure of FL. Furthermore, this study showed that patients who sequentially received PTX after FL and those treated with FLTAX had equivalent OS, highlighting the clinical importance of using both FU and PTX, either sequentially or in combination, for the patients with GC‐SPM.

Despite the benefits of using both medications, the selection of patient suitable for sequential strategy has remained a critical clinical concern. As such, we herein sought to identify baseline factors that would affect the likelihood of SUP after FL failure. Accordingly, our analysis identified two risk factors preventing SUP, namely GPS2 and initially unresectable disease. GPS is a well‐known prognostic factor in many cancer entities.^18^ Initially unresectable disease has worse impact on the survival of general metastatic GC.^9^ A plausible explanation is that periodic follow‐up surveys after surgical resection result in early detection of recurrence, which can cause a leading bias toward a better prognosis of recurrent disease than initially unresectable disease. Interestingly, both GPS2 and initially unresectable disease had been reported as a worse prognostic factor in GC‐SPM as shown in the subgroup analysis of the JCOG1108/WJOG7312G trial.^13^ As such, it can be reasonably assumed that poor general conditions would likely prevent the use of PTX after FL failure, thereby resulting in short survival. This study also attempted to demonstrate whether the SUP risk score can be used to select patients suitable for sequential treatment. Accordingly, our results demonstrated that an SUP risk score of 0 showed a high specificity (100%) but a very low sensitivity (13%) for identifying patients who can receive PTX after FL failure. After increasing the cutoff SUP risk score to 0–1, sensitivity increased to 67% but specificity decreased to 62%, indicating insufficient performance for prediction. These results suggest that the selection of patient suitable for the sequential strategy remains considerably difficult.

We further investigated differences in treatment efficacy between FL and FLTAX according to the SUP risk score. Notably, our findings showed that the SUP risk score was associated with the prognosis, with scores of 2, 1, and 0 representing worse, intermediate, and better OS, respectively. FLTAX showed a better OS compared to FL in patients with scores of 2 and 1, accounting for 90.4% (85/94) of those included in this study. Moreover, the superiority of FLTAX was more remarkable in the subgroup with a score of 2 and was modest in the subgroup with a score of 1. On the other hand, FL showed a better OS in patients with a score of 0, suggesting that FL may be favorable especially for patients with good general conditions. However, exceedingly few patients within GC‐SPM present with a good general condition, with only eight patients among those included herein having a SUP risk score of 0, which may be statistically underpowered to suggest the superiority of FL. Nonetheless, the SUP risk score may be used as an indicator for the selection of either FL or FLTAX, given the favorable outcomes of FLTAX in most of patients with GC‐SPM.

This study has several limitations worth noting. First, we excluded the FL/non‐PTX subgroup from this study given the unavailability of information on third‐line or later treatments. Therefore, we cannot rule out the possibility that outcomes of this subgroup could have affected the clinical implications of using FU and PTX presented herein. Second, patient number of each subgroup compared in this study was quite small. Thus, we did not conduct multivariate analyses to determine the association between two events. In particular, survival time comparison between FL/PTX and other subgroups included unadjusted and unbalanced patient background that definitely introduced bias into the results such that patients in the FL/PTX subgroup had better background compared to those in the FL/BSC or FLTAX subgroup, thereby favoring the FL/PTX subgroup in terms of survival. Likewise, the association between each factor and likelihood of SUP was not adjusted for other covariates, highlighting the need for careful interpretation of the results. Overall, due to the small sample size, we could not provide conclusive information. However, despite of being exploratory, it is important to deeply investigate the findings of the JCOG1108/WJOG7312G trial because it is so far the only randomized clinical trial that evaluates the treatment strategy of GC‐SPM. We believe the findings of this post hoc study would support the results of the JCOG1108/WJOG7312G trial suggesting FLTAX is a favorable treatment option in this population.

In conclusion, both the sequential and combined use of FU and PTX had been found to be useful in improving the survival of patients with GC‐SPM. However, the FLTAX combination regimen would be preferable given the difficulty in selecting patients suitable for sequential use.

## 5 Ethics approval and consent to participate

The review committees of the JCOG and WJOG and the institutional review boards of all participating institutions approved the study protocol of the JCOG1108/WJOG7312G trial (UMIN000010949), which was conducted according to the Declaration of Helsinki and Japanese Ethical Guidelines for Medical and Health Research Involving Human Subjects.

## CONFLICT OF INTEREST

EI reports personal fees from Pfizer Japan Inc., personal fees from Bristol Myers Squibb Company, personal fees from Nippontect Systems Co, Ltd., personal fees from RCR Co, Ltd., outside the submitted work; KY reports grants and personal fees from Taiho Pharmaceutical, personal fees from Chugai Pharm, personal fees from Bristol Myers Squibb Japan, personal fees from Merck Biopharm, personal fees from Takeda, grants from Yakult Honsha, grants from Sanofi, grants and personal fees from Ono Pharm, grants and personal fees from Elli Lilly, outside the submitted work; NB reports research fund from Ono and Takeda, and honorarium from Ono and Taiho; HH reports grants from AstraZeneca, grants and personal fees from Daiichi Sankyo, grants and personal fees from Sumitomo Dainippon Pharma, personal fees from Lilly, grants and personal fees from Merck Biopharma, grants and personal fees from MSD, grants and personal fees from Taiho, grants and personal fees from Chugai, grants from Eisai, grants from Elevar Therapeutics, grants from Incyte, grants from Pfizer, grants and personal fees from Boehringer ingelheim, grants from BeiGene, grants and personal fees from ONO, grants and personal fees from BMS, personal fees from Yakult Honsha, personal fees from Sanofi, personal fees from Takeda, grants from Astellas, personal fees from Kyowa Hakko Kirin, grants and personal fees from Bayel, grants from GSK, outside the submitted work; TN reports grants and personal fees from Taiho pharma, grants and personal fees from Chugai pharma, grants and personal fees from Ono pharma, grants from Bristol Myers Squibb, grants and personal fees from Daiichi Sankyo, grants from AstraZeneca, personal fees from takeda, personal fees from Lilly, grants from Dainippon Sumitomo, grants from MSD, outside the submitted work; KS1 reports grants and personal fees from Astellas Pharma, grants and personal fees from Eli Lilly and Company, personal fees from Bristol Myers Squibb, personal fees from Takeda Pharmaceuticals, personal fees from Pfizer Inc, grants and personal fees from Ono Pharmaceutical, personal fees from Novartis, personal fees from AbbVie Inc, personal fees from Yakult, grants from Dainippon Sumitomo Pharma, grants and personal fees from Daiichi Sankyo, grants and personal fees from Taiho Pharmaceutical, grants from Chugai Pharma, grants and personal fees from Merck Pharmaceutical, grants from Medi Science, personal fees from GlaxoSmithKline, personal fees from Amgen, personal fees from Boehringer Ingelheim, grants from Eisai, outside the submitted work; KS2 reports personal fees from Shionogi & Co., Ltd., personal fees from Takeda Pharmaceutical Co., Ltd., personal fees from Taiho Pharmaceutical Co., Ltd., personal fees from Ono Pharmaceutical Co., Ltd., personal fees from Merck Biopharma Co., Ltd., personal fees from Nippon Kayaku Co., Ltd., personal fees from Eisai Co., Ltd., personal fees from Bayer Yakuhin, Ltd., personal fees from Kyowa Hakko Kirin Co., Ltd., personal fees from Chugai Pharmaceutical Co., Ltd., personal fees from Eli Lilly Japan K.K., personal fees from Daiichi Sankyo Co., Ltd., outside the submitted work; IH reports grants and personal fees from Taiho Pharma, grants and personal fees from Chugai Pharma, grants and personal fees from Daiichi Sankyo Pharma, grants and personal fees from Yakult‐Honsha Pharma, grants and personal fees from Lilly, grants and personal fees from Takeda Pharma, grants and personal fees from Ono Pharma, during the conduct of the study; KM reports grants from Solasia Pharma, grants from Merck Serono, grants from Daiichi Sankyo, grants from Parexel International, grants from Pfizer, grants from MSD, grants and personal fees from Amgen, grants and personal fees from ONO Pharmaceutical CO.,LTD., grants and personal fees from Sanofi, grants and personal fees from Taiho, personal fees from AstraZeneca, personal fees from Chugai, personal fees from Takeda, personal fees from Eli Lilly, personal fees from Bristol Myers Squibb, personal fees from Bayer, outside the submitted work; MS reports personal fees from Taiho, personal fees from Daiichi Sankyo, personal fees from Chugai, personal fees from Ono, personal fees from Eli Lilly, personal fees from BMS Japan, outside the submitted work; MT2 reports personal fees from Taiho Pharmaceutical, personal fees from Chugai Pharmaceutical, personal fees from Ono Pharmaceutical, personal fees from BMS, personal fees from Yakult Honsha, personal fees from Takeda Pharmaceutical, personal fees from Eli Lilly Japan, personal fees from Pfizer Japan, personal fees from Daiichi Sankyo, personal fees from Johnson and Johnson, personal fees from Medtronic Japan, personal fees from Intuitive Japan, personal fees from Olympus, outside the submitted work; TEN reports grants from National Cancer Research and Development Fund (26‐A‐4, 29‐A‐3), grants from Grant‐in‐Aid for Clinical Cancer Research (H26‐144) from the Ministry of Health, Labour and Welfare of Japan, grants from Japan Agency for Medical Research and Development (AMED) (Grant Numbers JP16ck0106139 and JP18ck0106351), during the conduct of the study; grants and personal fees from Sumitomo Dainippon Pharma Co., personal fees from Boehringer Ingelheim, personal fees from Bristol Myers Squibb, grants and personal fees from Ono Pharmaceutical Co., grants and personal fees from Taiho Pharmaceutical Co., grants and personal fees from Amgen, grants and personal fees from Takeda Pharmaceutical Co., grants and personal fees from Chugai Pharmaceutical Co., grants and personal fees from Sanofi K.K., personal fees from Novartis Japan, grants and personal fees from Nippon Kayaku Co., grants and personal fees from MSD K.K., grants and personal fees from Eli Lilly Japan K.K., personal fees from Bayer Yakuhin, personal fees from Pfizer Japan Inc., grants and personal fees from Daiichi Sankyo Co., personal fees from Yakult Honsha Co., personal fees from Nipro Co, grants and personal fees from Merck Serono Co., personal fees from Celltrion Healthcare Japan, personal fees from Teijin Pharma, personal fees from Sawai Pharmaceutical Co., grants from Eisai Co, outside the submitted work; All remaining authors have declared no conflict of interest.

## AUTHOR'S CONTRIBUTIONS

HA and TEN planned, designed, and administered this study. The original draft was written by HA. EI was responsible for the statistical analysis. KY, NB, HH, TN, MT1, KS1, KS2, and SN made substantial contributions to data resources, investigations, and manuscript review and editing. IH, KM, MS, and MT2 supervised this study. All authors read and approved the final manuscript.

## Supporting information

Fig S1

Table S1

## Data Availability

The data used to support the findings of this study are available from the corresponding author upon request.
