# Supplementary material for: Clinical implications of using both fluoropyrimidine and paclitaxel in patients with severe peritoneal metastasis of gastric cancer: A post hoc study of JCOG1108/WJOG7312G
Source: Cancer Med. 2021 Oct 16;10(21):7673–82. doi: 10.1002/cam4.4303 (PMC8559492; doi:10.1002/cam4.4303)
Supplement: Supplementary file 1 — Fig S1 [file CAM4-10-7673-s001.pdf]

Figure S1

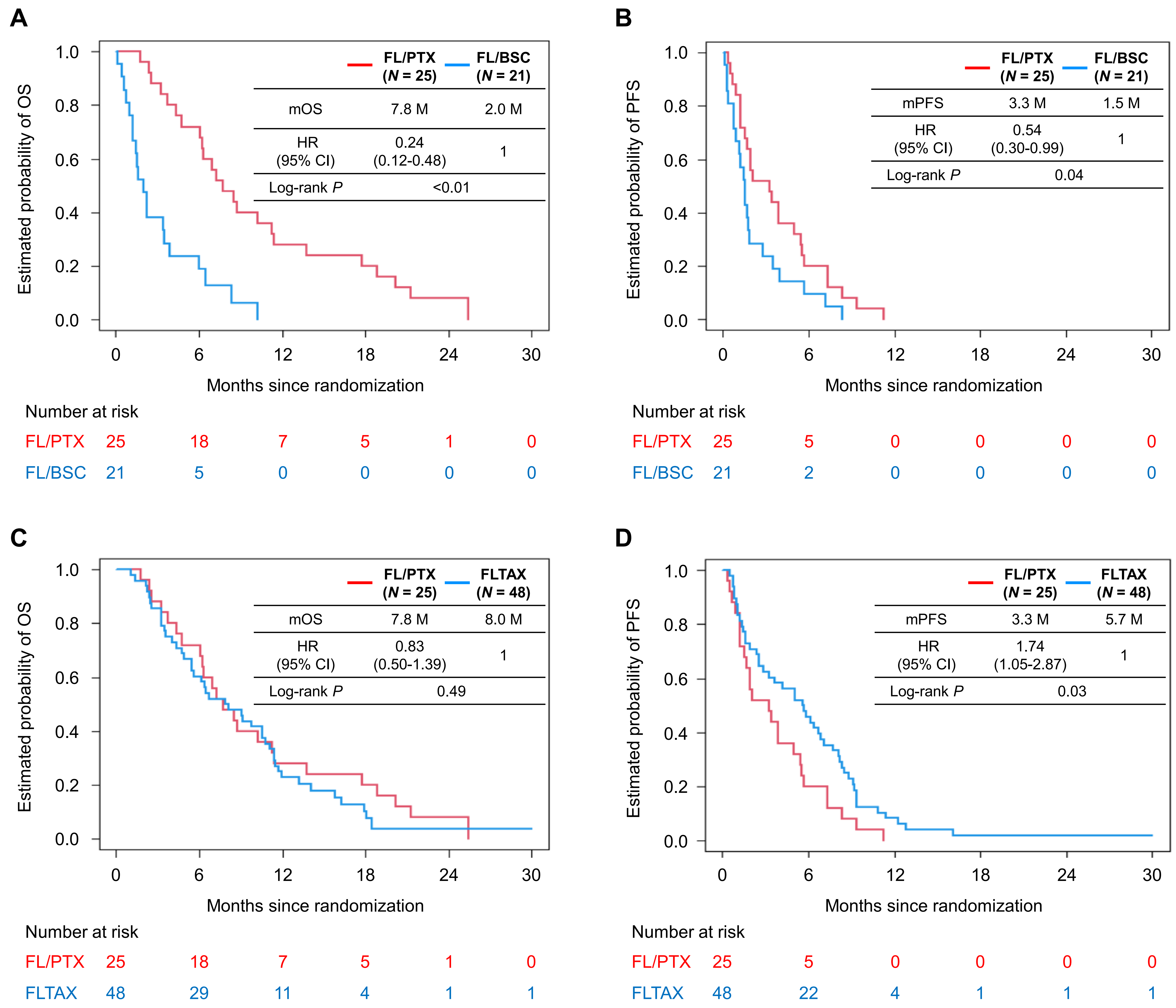

Kaplan-Meier curves comparing FL/PTX vs other subsets. (A) OS and (B) PFS between the FL/PTX and FL/BSC subsets; (C) OS and (D) PFS between the FL/PTX and FLTAX subsets.
Abbreviations: CI, confidence interval; HR, hazard ratio; mOS, median overall survival; mPFS, median progression-free survival; OS, overall survival; PFS, progression-free survival.
