# Supplementary material for: Clinical implications of using both fluoropyrimidine and paclitaxel in patients with severe peritoneal metastasis of gastric cancer: A post hoc study of JCOG1108/WJOG7312G
Source: Cancer Med. 2021 Oct 16;10(21):7673–82. doi: 10.1002/cam4.4303 (PMC8559492; doi:10.1002/cam4.4303)
Supplement: Supplementary file 2 — Table S1 [file CAM4-10-7673-s002.docx]

**Table S1. Patient characteristics at baseline stratified by the SUP risk score**

|  |  | SUP risk score 2 | | | | | SUP risk score 1 | | | | | SUP risk score 0 | | | | |
| --- | --- | --- | --- | --- | --- | --- | --- | --- | --- | --- | --- | --- | --- | --- | --- | --- |
|  |  | FL  (*N* = 21) | | FLTAX  (*N* = 21) | | *P* | FL  (*N* = 21) | | FLTAX  (*N* = 22) | | *P* | FL  (*N* = 3) | | FLTAX  (*N* = 5) | | *P* |
|  |  | *N* | (%) | *N* | (%) |  | *N* | (%) | *N* | (%) |  | *N* | (%) | *N* | (%) |  |
| Age | >65 | 8 | (38) | 9 | (43) | 0.26 | 11 | (52) | 15 | (68) | 0.29 | 1 | (33) | 2 | (40) | 0.85 |
|  | <65 | 21 | (62) | 12 | (57) |  | 10 | (48) | 7 | (32) |  | 2 | (67) | 3 | (60) |  |
| Sex | Male | 12 | (57) | 18 | (86) | 0.04 | 13 | (62) | 11 | (50) | 0.43 | 2 | (67) | 1 | (20) | 0.19 |
|  | Female | 9 | (43) | 3 | (14) |  | 8 | (38) | 11 | (50) |  | 1 | (33) | 4 | (80) |  |
| ECOG-PS | 0 | 0 | (0) | 2 | (10) | 0.23 | 5 | (24) | 5 | (23) | 0.68 | 0 | (0) | 0 | (0) | - |
|  | 1 | 14 | (67) | 10 | (48) |  | 10 | (48) | 13 | (59) |  | 3 | (100) | 5 | (100) |  |
|  | 2 | 7 | (33) | 9 | (43) |  | 6 | (29) | 4 | (18) |  | 0 | (0) | 0 | (0) |  |
| GPS | 0 | 0 | (0) | 0 | (0) | - | 5 | (24) | 7 | (32) | 0.77 | 2 | (67) | 4 | (80) | - |
|  | 1 | 0 | (0) | 0 | (0) |  | 13 | (62) | 13 | (59) |  | 1 | (33) | 1 | (20) |  |
|  | 2 | 21 | (100) | 21 | (100) |  | 3 | (14) | 2 | (9) |  | 0 | (0) | 0 | (0) |  |
| Disease status | Recurrent | 0 | (0) | 0 | (0) | - | 3 | (14) | 2 | (9) | 0.60 | 3 | (100) | 5 | (100) | - |
|  | Initially unresectable | 21 | (100) | 21 | (100) |  | 18 | (86) | 20 | (91) |  | 0 | (0) | 0 | (0) |  |
| No. of metastatic sites | 1-2 | 15 | (71) | 13 | (62) | 0.51 | 19 | (90) | 20 | (91) | 0.96 | 3 | (100) | 5 | (100) | - |
|  | >3 | 6 | (29) | 8 | (38) |  | 2 | (10) | 2 | (9) |  | 0 | (0) | 0 | (0) |  |
| Histological type | Differentiated | 5 | (24) | 2 | (10) | 0.25 | 6 | (29) | 2 | (9) | 0.12 | 0 | (0) | 1 | (20) | - |
|  | Undifferentiated | 15 | (71) | 19 | (90) |  | 15 | (71) | 18 | (82) |  | 3 | (100) | 4 | (80) |  |
|  | Others | 1 | (5) | 0 | (0) |  | 0 | (0) | 2 | (9) |  | 0 | (0) | 0 | (0) |  |
| Massive ascites | Present | 15 | (71) | 16 | (76) | 0.73 | 15 | (71) | 10 | (45) | 0.08 | 2 | (67) | 4 | (80) | 0.67 |
|  | Absent | 6 | (29) | 5 | (24) |  | 6 | (29) | 12 | (55) |  | 1 | (33) | 1 | (20) |  |
| Oral intake | Adequate | 10 | (48) | 10 | (48) | 1.00 | 13 | (62) | 8 | (36) | 0.09 | 1 | (33) | 4 | (80) | 0.19 |
|  | Inadequate | 11 | (52) | 11 | (52) |  | 8 | (38) | 14 | (64) |  | 2 | (67) | 1 | (20) |  |
| Subtype of SPM | Massive ascites | 10 | (48) | 10 | (48) | 0.91 | 13 | (62) | 8 | (36) | 0.21 | 1 | (33) | 4 | (80) | 0.29 |
|  | Inadequate oral intake | 6 | (29) | 5 | (24) |  | 6 | (29) | 12 | (55) |  | 1 | (33) | 1 | (20) |  |
|  | Both | 5 | (24) | 6 | (29) |  | 2 | (10) | 2 | (9) |  | 1 | (33) | 0 | (0) |  |

Abbreviations: ECOG-PS, Eastern Cooperative Oncology Group performance status; GPS, Glasgow Prognostic Score; SPM, severe peritoneal metastasis; SUP, sequential use of paclitaxel.
